# The first mitochondrial genome of the genus *Exhippolysmata* (Decapoda: Caridea: Lysmatidae), with gene rearrangements and phylogenetic associations in Caridea

**DOI:** 10.1038/s41598-021-93946-7

**Published:** 2021-07-14

**Authors:** Ying-ying Ye, Jing Miao, Ya-hong Guo, Li Gong, Li-hua Jiang, Zhen‑ming Lü, Kai-da Xu, Bao-ying Guo

**Affiliations:** 1 Marine Fishery Institute of Zhejiang Province, Key Laboratory of Sustainable Utilization of Technology Research for Fishery Resource of Zhejiang Province, Zhejiang Ocean University, Zhoushan, 316021 People’s Republic of China; 2 National Engineering Research Center for Marine Aquaculture, Zhejiang Ocean University, Zhoushan, 316022 Zhejiang People’s Republic of China; 3 National Engineering Laboratory of Marine Germplasm Resources Exploration and Utilization, Zhejiang Ocean University, Zhoushan, 316022 Zhejiang People’s Republic of China

**Keywords:** Biodiversity, Evolutionary genetics

## Abstract

The complete mitochondrial genome (mitogenome) of animals can provide useful information for evolutionary and phylogenetic analyses. The mitogenome of the genus *Exhippolysmata* (i.e., *Exhippolysmata ensirostris*) was sequenced and annotated for the first time, its phylogenetic relationship with selected members from the infraorder Caridea was investigated. The 16,350 bp mitogenome contains the entire set of 37 common genes. The mitogenome composition was highly A + T biased at 64.43% with positive AT skew (0.009) and negative GC skew (− 0.199). All tRNA genes in the *E. ensirostris* mitogenome had a typical cloverleaf secondary structure, except for *trnS1* (AGN), which appeared to lack the dihydrouridine arm. The gene order in the *E. ensirostris* mitogenome was rearranged compared with those of ancestral decapod taxa, the gene order of *trnL2*-*cox2* changed to *cox2*-*trnL2*. The tandem duplication-random loss model is the most likely mechanism for the observed gene rearrangement of *E. ensirostris*. The ML and BI phylogenetic analyses place all Caridea species into one group with strong bootstrap support. The family Lysmatidae is most closely related to Alpheidae and Palaemonidae. These results will help to better understand the gene rearrangements and evolutionary position of *E. ensirostris* and lay a foundation for further phylogenetic studies of Caridea.

## Introduction

The Decapoda is an ecologically and economically important order of crustaceans comprising a wide variety of crabs, lobsters, prawns and shrimps totalling over 18,000 extant and fossil species^1,2^. It is also the most abundant and largest order of crustaceans. Shrimps of the infraorder Caridea are commonly found in marine and freshwater habitats and have attracted attention due to their high commercial value^3–5^. Currently, there are 15 superfamilies recognized in the Caridea^6^. The family Lysmatidae is shown to comprise five genera, viz. *Lysmata* Risso, 1816; *Ligur* Sarato, 1885; *Mimocaris*, Nobili, 1903; *Lysmatella* Borradaile, 1915 and *Exhippolysmata* Stebbing, 1915. In the past, genetic studies of caridean families indicated that Hippolytidae is not a monophyletic taxa^7,8^ but should be partitioned into at least two families. Thereafter, morphological and genetic studies have recovered the Hippolytidae as polyphyletic, and the family Lysmatidae was formally resurrected^9^. Lysmatid shrimps are unique among crustaceans because of their enigmatic sexual system. They are protandric simultaneous hermaphrodites: shrimps initially mature and reproduce solely as males and later in life become functional simultaneous hermaphrodites^10^. In addition, due to their wide diversity of lifestyles, shrimp from the genus *Exhippolysmata* are particularly special.

The species *Exhippolysmata ensirostris* (Kemp 1914), which is widely distributed in the Pacific region, extends from the coast of the East China Sea and South China Sea to the Indo-West Pacific. It is an important and commercially exploited species in the East China Sea and the South China Sea. However, research on the genus *Exhippolysmata* has been limited to its species investigation and morphological description. Most of the research in Lysmatidae has focused on the genus *Lysmata*, including their mitochondrial genes and evolutionary relationships^11–16^. Consequently, research on the mitochondrial genes of the genus *Exhippolysmata* has rarely been reported.

The complete mitochondrial genome (mitogenome) is typically extrachromosomal and characterized by maternal inheritance and with a high evolution rate^17^. A complete mitogenome is a powerful tool for analysing the evolutionary history and phylogeny of species^18^. The mitogenome can also provide direct molecular clues for gene rearrangement processes, which would reveal important information for phylogenetic analyses^19^. The mitogenome of most metazoans is a double-stranded closed circular molecule approximately 11–20 kb in length. It typically contains 37 genes, including 13 protein coding genes (PCGs), two ribosomal RNA genes (*16S rRNA* and *12S rRNA*) and 22 transporter RNA genes^20^.

In this study, the first complete mitogenome of the genus *Exhippolysmata* was described for the first time. We first successfully determined the complete mitogenome sequence of *E. ensirostris* using Illumina sequencing technology. We also analysed the nucleotide composition, codon usage profiles of protein coding genes (PCGs), Ka/Ks ratios of 13 PCGs, tRNA secondary structures, gene order and investigate the evolutionary relationships within Caridea. The purpose of this study was to understand the characteristics of the *E. ensirostris* mitogenome and clarify the evolutionary relationships within the Caridea mitogenome.

## Results and discussion

### Genome organization and base composition

**Figure 1:**
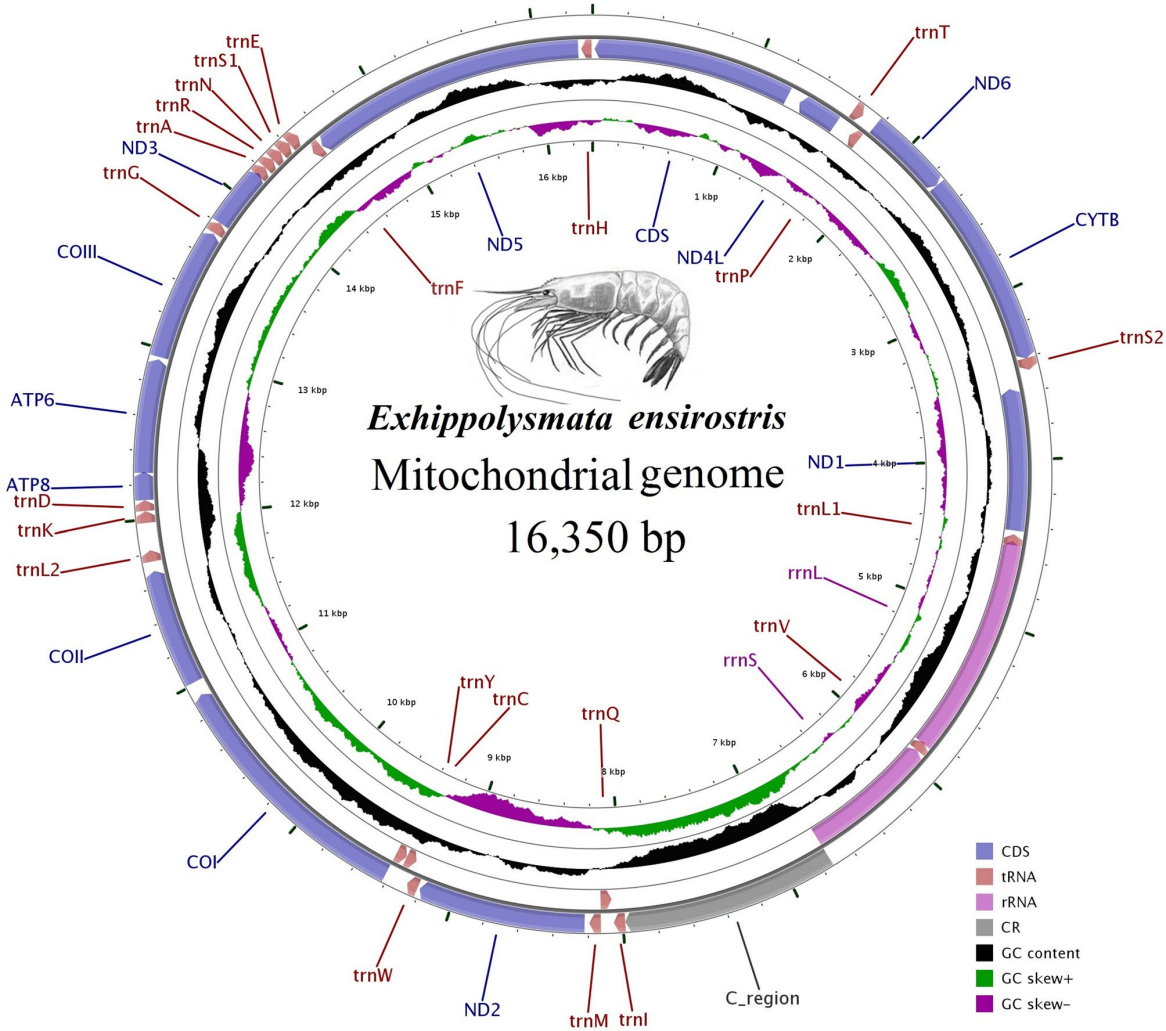
Circular mitogenome map of *Exhippolysmata ensirostris*. Protein coding, ribosomal and tRNA genes are shown with standard abbreviations. Arrows indicate the orientation of gene transcription. The inner circles show the GC content and GC skew, which are plotted as the deviation from the average value of the entire sequence.

The complete mitogenome of *E. ensirostris* was found to be a typical circular molecule of 16,350 bp (Fig. 1), and the sequence was deposited in GenBank under accession number MK681888. The data that support the findings of this study are openly available in Microsoft OneDrive at (https://1drv.ms/w/s!Ag1aKdaw8CT3iHxX9f98FCkZvQ3n?e=BaRfdq). The newly sequenced mitogenome contains 13 PCGs, 22 tRNA genes, two rRNA genes and a large noncoding or control region (CR). Of the 37 genes, 23 were encoded on the heavy strand, and the other 14 were encoded on the light strand (Fig. 1, Table 1). The longest noncoding region was located between *trnL2* and *trnK,* and the largest gene junction was located between *trnL1* and *12S rRNA*. The base compositions (Table 2) showed a high A + T content in the complete mitogenome (64.43%), PCGs (62.6%), tRNAs (66.04%), rRNAs (66.62%) and a CR (69.33%). The relative order of the nucleotide composition was A > T > C > G. The complete sequence had a positive AT skew (0.009) and a negative GC skew (− 0.199). As in other invertebrate mtDNAs, there were overlapping and noncoding bases between some genes.

**Table 1:** Organization of the *Exhippolysmata ensirostris* mitochondrial genome.

Gene	Direction	Position	Length (bp)	Anticodon	Start codon	Stop codon
*nad4*	−	13–1239	1227	−	ATG	TAA
*nad4l*	−	1323–1577	255	−	ATG	TAA
*tRNA-Thr* (T)	+	1618–1680	63	ACA	−	−
*tRNA-Pro* (P)	−	1686–1748	63	CCA	−	−
*nad6*	+	1773–2270	498	−	ATA	TAA
*cob*	+	2272–3408	1137	−	ATG	TAA
*tRNA-Ser2*	+	3407–3476	70	TCA	−	−
*nad1*	−	3564–4433	870	−	ATA	TAG
*tRNA-Leu1*	−	4455–4521	67	CTA	−	−
*16S rRNA*	−	4499–5866	1368	−	−	−
*trnV*	−	5858–5921	64	GTA	−	−
*12S rRNA*	−	5920–6737	818	−	−	−
*CR*	+	6738–7986	1249	−	−	−
*tRNA-Ile* (I)	+	7987–8053	67	ATC	−	−
*tRNA-Gln* (Q)	−	8060–8127	68	CAA	−	−
*tRNA-Met* (M)	+	8129–8195	67	ATG	−	−
*nad2*	+	8223–9204	982	−	ATG	TAA
*tRNA-Trp* (W)	+	9219–9284	66	TGA	−	−
*tRNA-Cys* (C)	−	9288–9352	65	TGC	−	−
*tRNA-Tyr* (Y)	−	9354–9418	65	TAC	−	−
*cox1*	+	9420–10,931	1512	−	ATA	TAA
*cox2*	+	11,013–11,699	687	−	ATG	TAA
*tRNA-Leu2*	+	11,756–11,821	66	TTA	−	−
*tRNA-Lys* (K)	+	11,981–12,048	68	AAA	−	−
*tRNA-Asp* (D)	+	12,050–12,112	63	GAC	−	−
*atp8*	+	12,113–12,277	165	−	ATG	TAA
*atp6*	+	12,271–12,936	666	−	ATG	TAA
*cox3*	+	12,951–13,733	783	−	ATA	TAA
*tRNA-Gly* (G)	+	13,740–13,806	67	GGA	−	−
*nad3*	+	13,807–14,172	366	−	ATG	TAA
*tRNA-Ala* (A)	+	14,159–14,221	63	GCA	−	−
*tRNA-Arg* (R)	+	14,222–14,285	64	CGA	−	−
*tRNA-Asn* (N)	+	14,285–14,349	65	AAC	−	−
*tRNA-Ser1*	+	14,350–14,417	68	AGA	−	−
*tRNA-Glu* (E)	+	14,418–14,486	69	GAA	−	−
*tRNA-Phe* (F)	−	14,487–14,550	64	TTC	−	−
*nad5*	−	14,558–16,261	1704	−	ATG	TAA
*tRNA-His* (H)	−	16,280–16,343	64	CAC	−	−

**Table 2:** Nucleotide composition and skewness of the *Exhippolysmata ensirostris* mitochondrial genome.

*E. ensirostris*	Size (bp)	A%	T%	G%	C%	A + T%	AT-skew	GC-skew
Mitogenome	16,350	32.51	31.91	14.24	21.33	64.43	0.009	− 0.199
*nad4*	1227	22.96	39.82	23.05	14.17	62.79	− 0.269	0.239
*nad4l*	255	23.53	38.43	25.88	12.16	61.96	− 0.240	0.361
*nad6*	498	27.91	36.55	12.65	22.89	64.46	− 0.134	− 0.288
*cob*	1137	26.47	35.09	15.92	22.52	61.57	− 0.140	− 0.172
*nad1*	870	22.76	40.34	23.33	13.56	63.1	− 0.279	0.265
*nad2*	982	26.48	36.25	10.9	26.37	62.73	− 0.156	− 0.415
*cox1*	1512	26.32	33.8	17.99	21.89	60.12	− 0.124	− 0.098
*cox2*	687	31	32.61	15.87	20.52	63.61	− 0.025	− 0.128
*atp8*	165	34.55	41.82	4.85	18.79	76.36	− 0.095	− 0.590
*atp6*	666	26.43	36.04	12.91	24.62	62.46	− 0.153	− 0.312
*cox3*	783	2682	33.59	17.5	22.09	60.41	0.975	− 0.116
*nad3*	366	26.78	37.43	13.93	21.86	64.21	− 0.166	− 0.222
*nad5*	1704	25.23	38.32	22.77	13.67	63.56	− 0.206	0.250
PCGs	10,852	26	36.6	18	19.4	62.6	− 0.169	− 0.037
tRNAs	1446	32.92	32.13	18.74	15.21	66.04	0.012	0.104
rRNAs	2186	31.05	35.57	20.53	12.85	66.62	− 0.068	0.230
CR	1249	35.23	34.09	14.14	16.53	69.33	0.016	− 0.078

### Protein coding genes and noncoding regions

**Figure 2:**
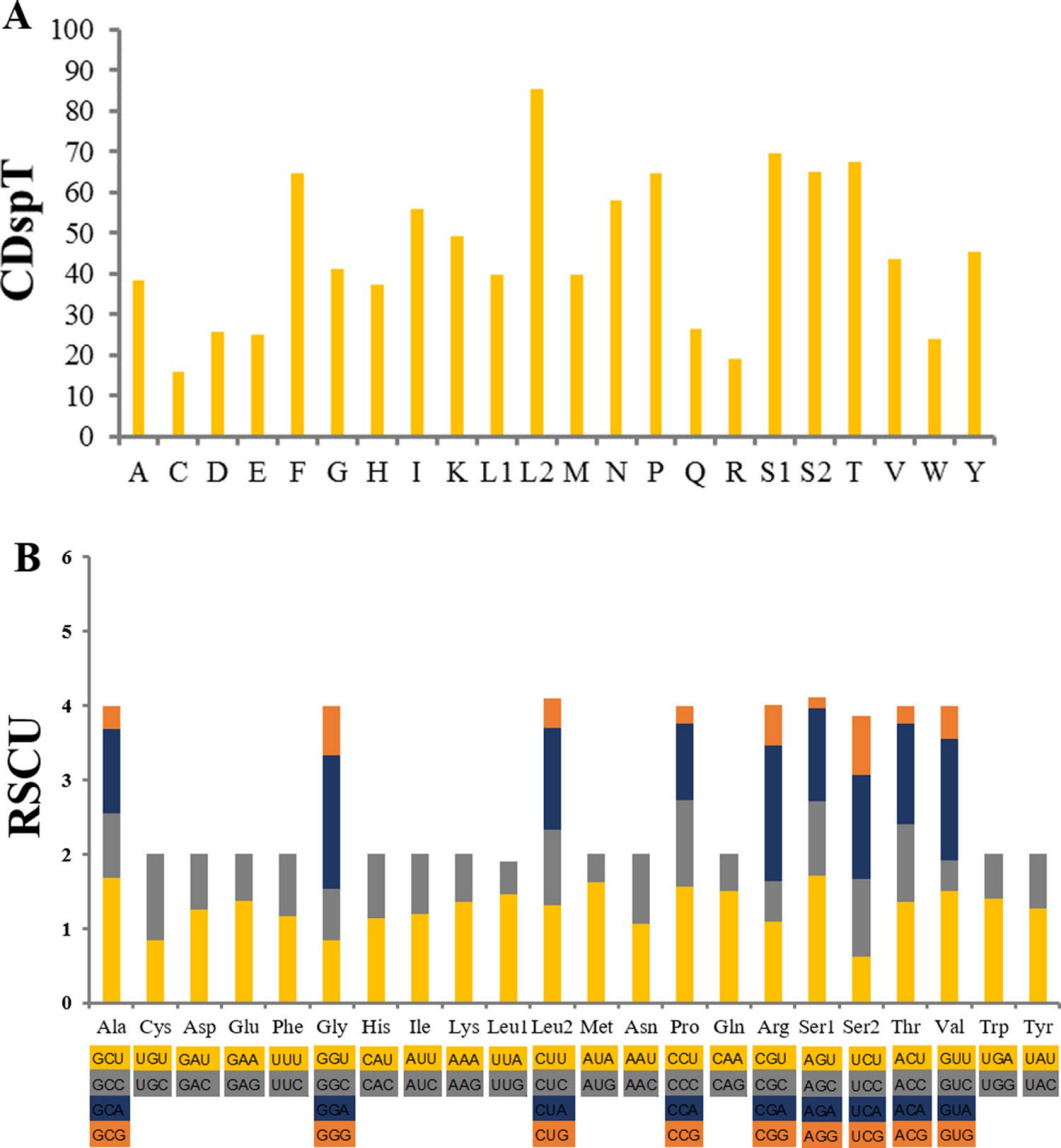
Codon usage patterns in the mitogenome of *Exhippolysmata ensirostris* CDspT, codons per thousand codons. Codon families are provided on the x axis (**A**); the relative synonymous codon usage (RSCU) (**B**).

The total length of the 13 PCGs was 10,852 bp and accounted for 66.3% of the whole *E. ensirostris* mitogenome. The 13 PCGs ranged from 165 bp (*ATP8*) to 1704 bp (*nad5*) (Tables 1, 2). Nine PCGs (*cox1*, *cox2*, *cox3*, *nad2*, *nad3*, *nad6*, *atp8*, *atp6*, *and cob*) were encoded on the heavy strand, and the other four PCGs (*nad5*, *nad4*, *nad4 L* and *nad1*) were encoded on the light strand (Table 1). Three genes (*nad6*, *cox1* and *cox3*) were found to start with ATA, a further three (*nad5*, *nad4* and *nad4 L*) with ATT, and the other seven with ATG. Eleven PCGs were found to end with the typical stop codon TAA, whereas *cox1* and *nad4* were found to end with TAG. Codon number and relative synonymous codon usage in the *E. ensirostris* mitochondrial genome are listed in Table 3. The patterns of codon usage of 13 PCGs are exhibited in Fig. 2A. The abundance of codon families and the relative synonymous codon usage (RSCU) in the PCGs were investigated for all available *E. ensirostris* mtDNAs, and the results are shown in Fig. 2B. The most frequently used codon was UUR (*trnL2*). There were 22 non-coding regions and eight overlaps of neighbouring genes in the mitochondrial genome of *E. ensirostris*. The largest non-coding region of *E. ensirostris* was identified as a putative control region. In addition, the position of the largest gene overlap (23 bp) was between *trnL1* and *16S rRNA*.

**Table 3:** Codon number and relative synonymous codon usage in the *Exhippolysmata ensirostris* mitochondrial genome.

Codon	Count	RSCU	Codon	Count	RSCU	Codon	Count	RSCU	Codon	Count	RSCU
UUU (F)	132	1.17	UCU (S)	101	1.72	UAU (Y)	100	1.27	UGU (C)	23	0.84
UUC (F)	93	0.83	UCC (S)	58	0.99	UAC(Y)	58	0.73	UGC (C)	32	1.16
UUA (L)	106	1.46	UCA (S)	74	1.26	UAA (*)	99	1.51	UGA(W)	59	1.4
UUG (L)	32	0.44	UCG (S)	9	0.15	UAG (*)	32	0.49	UGG(W)	25	0.6
CUU (L)	95	1.31	CCU (P)	88	1.56	CAU (H)	74	1.14	CGU (R)	18	1.09
CUC (L)	74	1.02	CCC (P)	66	1.17	CAC (H)	56	0.86	CGC (R)	9	0.55
CUA (L)	99	1.37	CCA (P)	58	1.03	CAA (Q)	69	1.5	CGA (R)	30	1.82
CUG (L)	29	0.4	CCG (P)	13	0.23	CAG (Q)	23	0.5	CGG (R)	9	0.55
AUU (I)	117	1.2	ACU (T)	80	1.36	AAU (N)	107	1.06	AGU (S)	37	0.63
AUC (I)	78	0.8	ACC (T)	61	1.04	AAC (N)	95	0.94	AGC (S)	61	1.04
AUA (M)	112	1.62	ACA (T)	80	1.36	AAA (K)	117	1.36	AGA (S)	82	1.4
AUG (M)	26	0.38	ACG (T)	14	0.24	AAG (K)	55	0.64	AGG (S)	47	0.8
GUU (V)	57	1.5	GCU (A)	56	1.68	GAU (D)	56	1.26	GGU (G)	30	0.84
GUC (V)	16	0.42	GCC (A)	29	0.87	GAC (D)	33	0.74	GGC (G)	25	0.7
GUA (V)	62	1.63	GCA (A)	38	1.14	GAA (E)	60	1.38	GGA (G)	64	1.79
GUG (V)	17	0.45	GCG (A)	10	0.3	GAG (E)	27	0.62	GGG (G)	24	0.67

**Figure 3:**
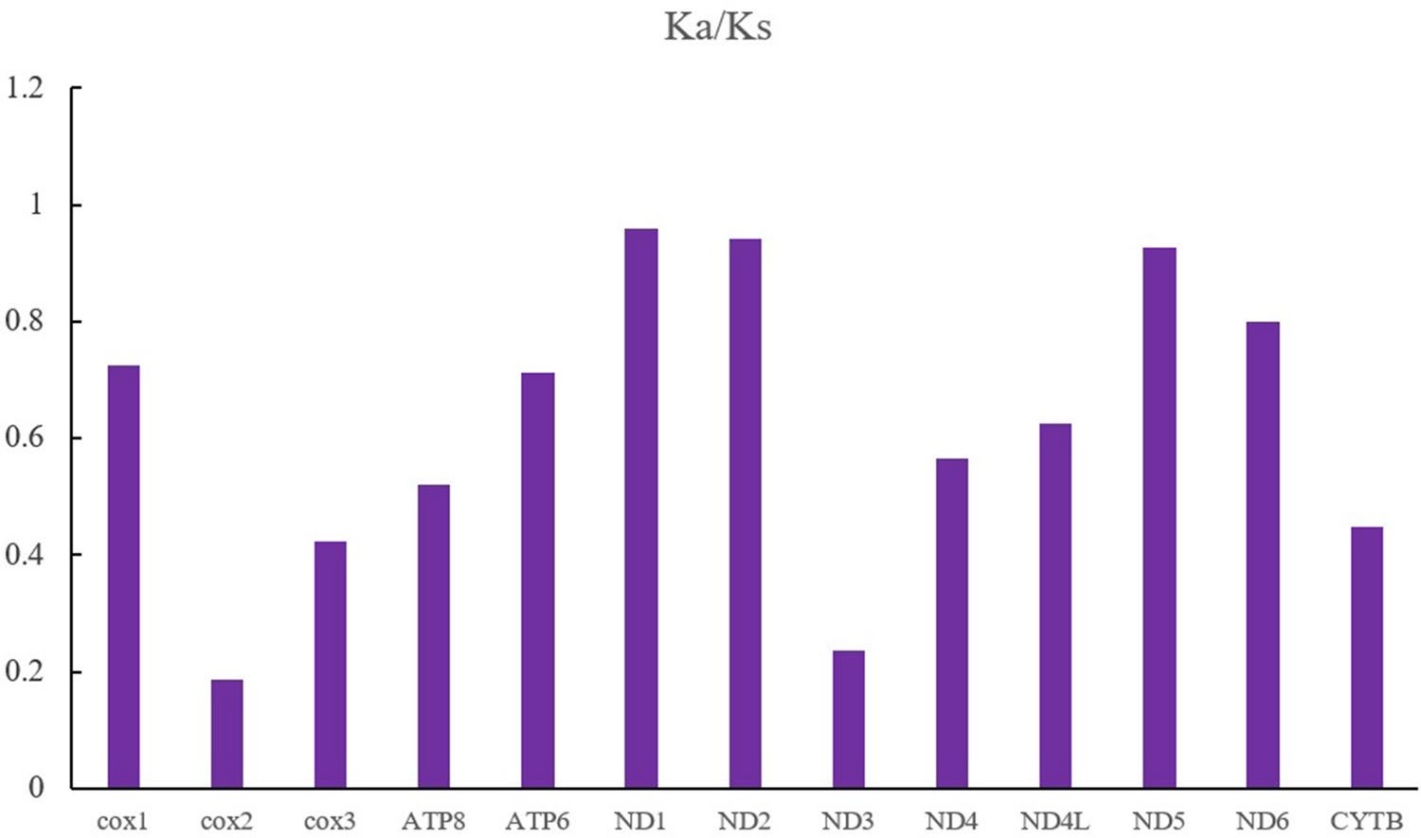
The ratio of synonymous and nonsynonymous substitution rates (Ka/Ks) in all 13 mitochondrial PCGs of seven caridean shrimp. Ka: nonsynonymous substitution rate; Ks: synonymous substitution rate. The histogram represents the average Ka/Ks for each PCG.

To analyse the selection pressure on mitochondrial PCGs of the caridean shrimps, the ratio of the nonsynonymous and synonymous substitution rates (Ka/Ks) for the 13 PCGs from the six caridean species (*E. ensirostris*, *Alpheus japonicas*, *Alvinocaris longirostris*, *Halocaridina rubra*, *Heterocarpus ensifer* and *Macrobrachium lanchesteri*) was calculated. We found that the Ka/Ks values for all PCGs were lower than one (between 0.187 and 0.959), indicating that they are evolving under purifying selection (Fig. 3). Among all 13 caridean protein-coding genes, the average Ka/Ks of *nad1* was the highest (0.959), and *nad2* (0.941) and *nad5* (0.927) also had very high average Ka/Ks values, indicating that these genes bear less selective pressure than other mitochondrial protein-coding genes.

### Transfer and ribosomal RNA genes

**Figure 4:**
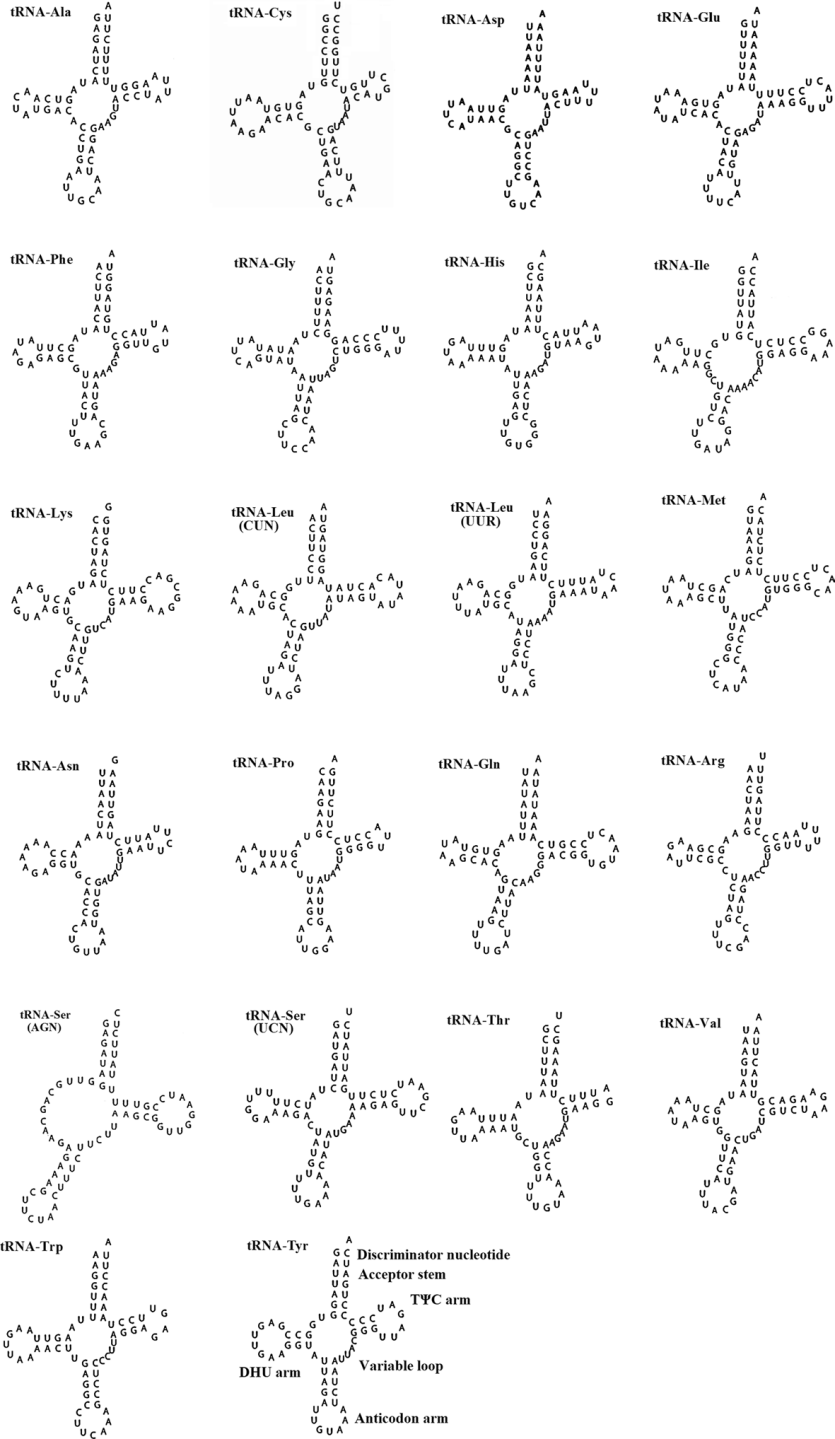
Putative secondary structures of tRNAs from the *Exhippolysmata ensirostris* mitogenome. The tRNAs are labelled with the abbreviations of their corresponding amino acids.

The *E. ensirostris* mitochondrial genome encodes 22 tRNA genes, each of which was predicted to fold into a clover-leaf secondary structure that ranged in size from 64 bp (*trnC*) to 70 bp (*trnV*) of nucleotides (Table 1). The DHU arm of the *trnS1* gene lacked any secondary structure (Fig. 4). The total length of the 22 tRNA genes in the *E. ensirostris* mitochondrial genome was 1446 bp. The overall A + T content of tRNA genes was 66.04%, which is similar to that of other carideans (Table 2)^21^. The mt tRNAs had a weakly positive AT skew (0.012) and positive GC skew (0.104). Fourteen tRNA genes (*trnL2*, *trnK*, *trnD*, *trnG*, *trnA*, *trnR*, *trnN*, *trnS1*, *trnE*, *trnT*, *trnS2*, *trnI*, *trnM* and *trnW)* were present on the heavy strand, and eight tRNA genes (*trnF*, *trnH*, *trnP*, *trnL1*, *trnV*, *trnQ*, *trnC* and *trnY*) were present on the light strand.

The *12S rRNA* gene lay between *trnL1* (CUN) and *trnV*, while the *16S rRNA* gene lay between *trnV* and the putative control region, and both rRNA genes were encoded by the β-strand. As typically seen in other shrimp mitogenomes, the *16S rRNA* and *12S rRNA* genes of the *E. ensirostris* mitogenome were 1368 bp and 818 bp in length, respectively. The location and orientation of the rRNA genes were identical to the original arrangement of ancestral Caridea^22^. The A + T content of the two rRNA genes was 66.62%, and they had a negative AT skew (− 0.068, Table 2).

### Gene rearrangement

**Figure 5:**
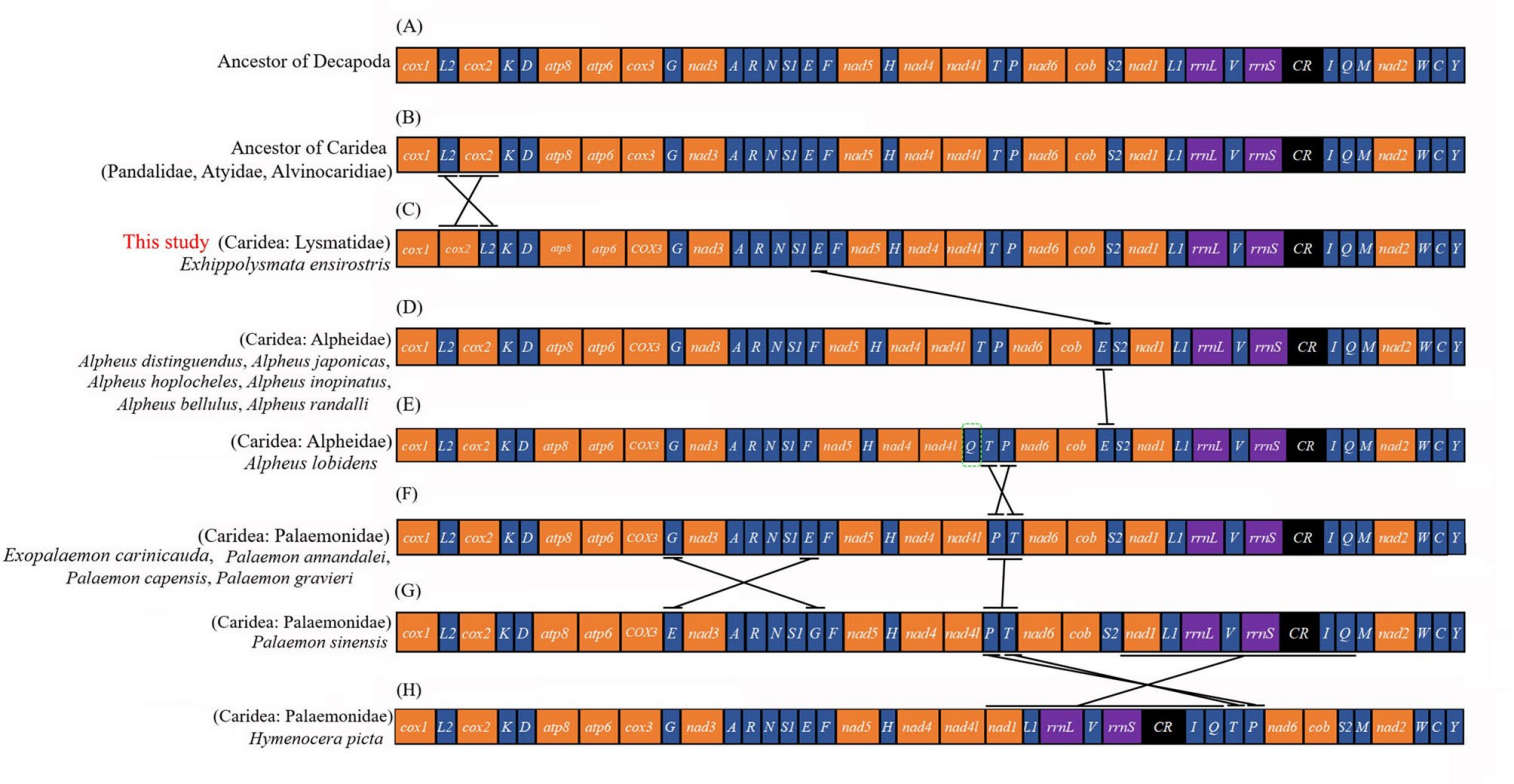
Linear representation of gene arrangements of an (**A**) ancestor of Decapoda; (**B**) ancestor of Caridea; (**C**) the Lysmatidae species *Exhippolysmata ensirostris*; (**D**) the Alpheidae species *Alpheus distinguendus*, *Alpheus hoplocheles*, *Alpheus inopinatus*, *Alpheus bellulus*, *Alpheus randalli* and *Alpheus japonicas*; (**E**) the Alpheidae species *Alpheus lobidens*; (**F**) the Palaemonidae species *Exopalaemon carinicauda*, *Palaemon annandalei*, *Palaemon capensis* and *Palaemon gravieri*; (**G**) the Palaemonidae species *Palaemon sinensis*; (**H**) the Palaemonidae species *Hymenocera picta*. All genes are transcribed from left to right. The green box indicated the duplicated gene. *16S rRNA* and *12S rRNA* are the large and small ribosomal RNA subunits, respectively.

Gene rearrangement in the Decapoda mitogenome commonly occurs and can be a tool to study phylogenetic relationships. Tan et al.^19^ gave an overview of mitochondrial gene orders (MGOs) of Decapoda, which revealed a large number of MGOs deviating from the ancestral arthropod ground pattern and unevenly distributed among infraorders. Here, we compared the MGOs of the Caridea mitogenomes with ancestral Decapoda and Caridea (Fig. 5). Among them, the MGOs in the mitogenomes of the families Pandalidae, Atyidae, and Alvinocarididae were identical to those of the ancestral Decapoda. However, fourteen carideans from the families of Lysmatidae, Alpheidae and Palaemonidae displayed gene rearrangements. This is in contrast with previous views that the gene order in Caridea is conserved^23–26^. Compared with the gene order of the ancestral Decapoda, *E. ensirostris* has a translocation, for which the gene order is *trnL2-cox2* instead of *cox2-trnL2* (Fig. 5C). *Alpheus distinguendus*, *Alpheus hoplocheles*, *Alpheus inopinatus*, *Alpheus bellulus*, *Alpheus randalli* and *Alpheus japonicas* in Alpheidae also undergo gene rearrangement, and *trnE* translocates and reverses with *trnP*^27^ (Fig. 5D). *Alpheus lobidens* has an extra duplication of *trnQ* located downstream of *nad4l*^28^ (Fig. 5E). In addition, the translocation of two tRNA genes was found in the mitochondrial genomes of *Exopalaemon carinicaud*a, *Palaemon annandalei*, *Palaemon capensis* and *Palaemon gravieri* in Palaemonidae*,* wherein *trnP* or *trnT* were translocated, while the arrangement of other genes was identical^29^ (Fig. 5F). *Palaemon sinensis* in Palaemonidae has an extra translocation between trnG and *trnE* (Fig. 5G). The mitochondrial genome of *Hymenocera picta* in Palaemonidae bears a novel gene order, the gene block (*nad1-trnL1-16S rRNA- trnV-12S rRNA-CR- trnI- trnQ*) was rearranged from the downstream of *trnS2* to the position downstream of *nad4l* (Fig. 5H)*.* These data indicate that gene order is not conserved among caridean shrimp and could be useful for inferring phylogenetic relationships within Caridea when more mitochondrial data from Caridea become available in the future.

Some mechanisms have been proposed to explain the rearrangement of genes in animal mitogenomes, including the tandem duplication/random loss model (TDRL)^30^, tandem duplication/non-random loss model (TDNL)^31^, and recombination^32^. Generally, TDRL is one of the most widely accepted mechanisms of mitochondrial gene rearrangement, which involves tandem duplication of gene regions caused by downstream chain mismatch during replication. TDNL attribute gene rearrangement to clustering by common polarity. The recombination within mitochondria mechanism involves the breaking and reconnecting of DNA double strands, leading to gene rearrangement and gene inversion^33^. Here, we propose that TDRL is more capable of explaining the *cox2* and *trnL2* translocations of the tRNA genes in the *E. ensirostris* mitochondrial genome.

### Phylogenetic relationships

**Figure 6:**
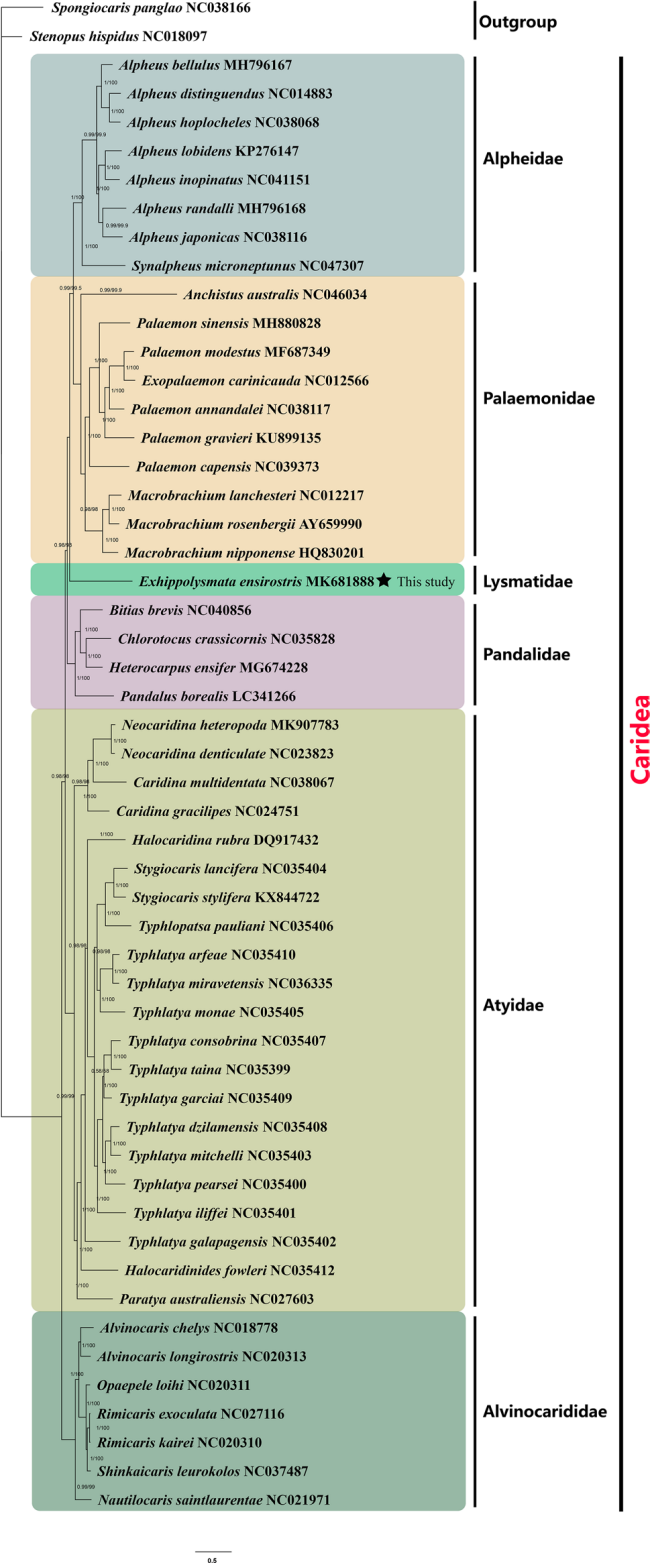
The phylogenetic tree was inferred from the nucleotide sequences of 13 mitogenome PCGs using BI and ML methods. Numbers on branches indicate posterior probability (BI) and bootstrap support (ML).

Many studies on the classification and evolutionary history of the Decapoda relied on morphological characteristics, which led to conflicting phylogenetic relationships. Under the best model, both ML and BI analyses of two data sets, based on the nucleotide sequences of the 13 PCGs and reconstruction of 53 species (including 51 Caridea species and two outgroup species) revealed the phylogenetic relationship between them. This study proposes a consistent phylogenetic relationship based on BI and ML methods; therefore, only one phylogenetic tree with both support values is presented (Fig. 6). Our results indicate that the mitochondrial genome sequence is robust for the inference of the relationships between shrimps. In addition, both ML and BI analyses of the two data sets show high branch support values. The phylogenetic tree based on the mitogenomes indicates that Palaemonidae and Alpheidae forme a monophyletic group and show a statistically significant relationship at the family level. Our complete mitogenome data suggest phylogenetic relationships among the major lineages of Caridea as ((((Alpheidae + Palaemonidae) + Lysmatidae) + Pandalidae) + Atyidae) + Alvinocarididae.

Although the main phylogenetic structures of our tree were consistent with those of previous result, some controversial findings were observed. Here, the families Alpheidae, Pandalidae, Lysmatidae and Palaemonidae clustered together as sister groups and were distantly related to Alvinocarididae, which supports the previous finding revealed by five nuclear genes (18S, Enolase, H3, NaK and PEPCK) in Li et al.^8^. However, Li et al.^8^ also revealed that Atyidae has been considered as basal lineages within the Caridea, which was conflict with our results. Based on both mitochondrial and nuclear genes (16S and 18S), Bracken et al. also revealed Atyidae represent basal lineages within the Caridea^7^. Meanwhile, in Sun et al.’s recent study, the phylogenetic relationship among Caridea was ((((Alpheidae + Palaemonidae) + Pandalidae) + Alvinocarididae) + Atyidae), which also considered Atyidae was distantly related to the four above families^34^. Furthermore, our result does not agree with Tan et al.^35^ and Wang et al.^28^, which state that Atyidae was the sister clade to Alvinocarididae. In our phylogenetic tree, most of the unstable and conflicting clades might have resulted from the limited taxon samples. The sequencing and assembly of the mitochondrial genome current result will promote the future work of further mitochondrial genome sequencing, and to increase in taxon sampling and genome sequencing which will help to resolve the classification of Caridea. Thus, more mitochondrial genome data will lead to a more comprehensive understanding of the phylogenetic relationships within Caridea and to resolve its classification.

## Conclusions

Using next-generation sequencing methods, the mitogenome of *E. ensirostris* was determined to be a circular molecule of 16,350 bp. Compared with typical Decapoda mitogenomes, the gene order of this species had undergone a rearrangement, wherein *cox2* and *trnL2* were translocated to *trnL2* and *cox2.* The gene rearrangement event occurring in *E. ensirostris* mitogenome can be explained by the TDRL model. The evolutionary patterns of PCGs were observed in the six caridean shrimp mitogenomes, which indicates that these genes were evolving under purifying selection. Phylogenetic analyses indicated the Caridea clades as monophyletic groups with strong bootstrap support. The family Lysmatidae is most closely related to Alpheidae and Palaemonidae. However, the lack of complete mitogenomes of other species of the Lysmatidae has limited the understanding of the evolution of this group at the genome level. Therefore, further studies are required to elucidate the phylogenetic status of species belonging to this group and their relationships.

## Materials and methods

### Sampling, identification and DNA extraction

An individual specimen of *E. ensirostris* was collected from Zhoushan, Zhejiang Province, China (30° 09′ 41″ N, 122° 35′ 10″ E) by bottom trawl fishery resource monitoring in November 2018. The specimen was identified morphologically and preserved in absolute ethanol. The total genomic DNA was extracted from muscle tissues of the specimen by the salt-extraction procedure with a slight modification^36^. Once extracted, the DNA was stored in 1 × TAE buffer at 4 °C. The extracted DNA was identified by 1.5% agarose gel electrophoresis and stored at − 20 °C.

### Sequencing and assembly

The mitogenome of *E. ensirostris* was sequenced using next-generation sequencing by Origin Gene Co. Ltd., Shanghai, China. The mitogenome was sequenced from the total genomic DNA using an Illumina HiSeq X Ten platform to generate a library with an insert size of 400 bp. Then, the raw image data were converted into sequential data by base calling. A total of 5,515,049,137 bp of clean data and 37,141,698 clean reads were retrieved. Raw sequencing data were deposited into the Sequence Read Archive (SRA) database (SRR12199494) (http://www.ncbi.nlm.nih.gov/Traces/sra). De novo assembly of clean data without sequencing adapters was conducted using NOVOPlasty software (https://github.com/ndierckx/NOVOPlasty)^37^.

### Mitochondrial genome annotation and analysis

Based on the sequence of the complete de novo assembled mitochondrial genome set, MITOS tools (http://mitos2.bioinf.uni-leipzig.de/index.py) was used for annotation with manual correction^38^. To ensure the accuracy of the assembled mitogenome, we first compared it to those of other Lysmatidae species and then further verified it using NCBI BLAST searches of the *cox1* barcode sequence^39^. Base composition and relative synonymous codon usage (RSCU) values were calculated using MEGA v. 7.0^40^. Identification of tRNA genes was verified using the MITOS WebServer. The rRNA genes were determined based on the locations of adjacent tRNA genes and by comparisons with other shrimp. Strand asymmetry was calculated using the formulae AT-skew = (A − T)/(A + T) and GC-skew = (G − C)/(G + C)^41^. The graphical map of the circular *E. ensirostris* mitogenome was drawn using the online mitochondrial visualization tool CGView Server^42^. In addition, we estimated the value of synonymous (Ks) and nonsynonymous substitutions (Ka) in the 13 mitochondrial PCGs using DnaSP 5.1.0^43^. A Ka/Ks rate that is significantly less than one indicates negative (purifying) selective pressure, and a Ka/Ks rate that is significantly greater than 1 indicates positive selection^44^.

### Phylogenetic analysis

A total of 51 caridean shrimp mitogenomes were downloaded from GenBank (https://www.ncbi.nlm.nih.gov/genbank/) for phylogenetic analysis (Table 4). The outgroup taxa were two Stenopodidea species: *Stenopus hispidus* and *Spongiocaris panglao*. We used the nucleotide sequences of the 13 protein coding genes (PCGs) to construct ML and BI phylogenetic trees. The 13 mitochondrial PCGs were aligned through MAFFT using default settings^45^, and then the resulting alignments were imported into Gblocks v. 0.91b (http://molevol.cmima.csic.es/castresana/Gblocks_server.html) to select the conserved regions^46^. A substitution saturation analysis was performed in DAMBE v. 5.3.15 to test whether the dataset was suitable for constructing trees^47^. ML analysis was conducted using IQ-TREE v1.4.1^48^ with the best-fit substitution model automatically selected by ModelFinder^49^ in the IQ-TREE package. GTR + I + G was selected as the best-fit model for nucleotide datasets under the Akaike Information Criterion (AIC) by MrModeltest 2.3^50^, and then BI analysis was carried out using MrBayes 3.2.6^51^ BI analysis was performed using default settings over four independent runs for 2 million generations sampled every 100 generations. The average standard deviation of split frequencies was < 0.01, the estimated sample size was > 200 and the potential scale reduction factor approached 1.0. The first 25% of samples were discarded as burn-in, and the remaining trees were used to calculate the Bayesian posterior probabilities for a 50% majority-rule consensus tree. All parameters were checked with Tracer v. 1.6 (http://tree.bio.ed.ac.uk/software/tracer/). The resulting phylogenetic trees were visualized in FigTree v. 1.4.4 (http://tree.bio.ed.ac.uk/software/figtree/).

**Table 4:** Classification and mitochondrial genome information of families from Caridea.

Order	Family	Species	Size (bp)	Accession no
Caridea	Alpheidae	*Alpheus bellulus*	15,738	MH796167
Caridea	Alpheidae	*Alpheus distinguendus*	15,700	NC014883
Caridea	Alpheidae	*Alpheus hoplocheles*	15,735	NC03868
Caridea	Alpheidae	*Alpheus inopinatus*	15,789	NC041151
Caridea	Alpheidae	*Alpheus japonicus*	16,619	NC038116
Caridea	Alpheidae	*Alpheus lobidens*	15,735	KP276147
Caridea	Alpheidae	*Alpheus randalli*	15,676	MH796168
Caridea	Alpheidae	*Synalpheus microneptunus*	15,603	NC047307
Caridea	Alvinocarididae	*Alvinocaris chelys*	15,910	NC018778
Caridea	Alvinocarididae	*Alvinocaris longirostris*	16,050	NC020313
Caridea	Alvinocarididae	*Nautilocaris saintlaurentae*	15,928	NC021971
Caridea	Alvinocarididae	*Rimicaris exoculata*	15,902	NC027116
Caridea	Alvinocarididae	*Rimicaris kairei*	15,900	NC020310
Caridea	Alvinocarididae	*Shinkaicaris leurokolos*	15,903	NC037487
Caridea	Alvinocarididae	*Opaepele loihi*	15,905	NC020311
Caridea	Atyidae	*Caridina gracilipes*	15,550	NC024751
Caridea	Atyidae	*Caridina multidentata*	15,825	NC038067
Caridea	Atyidae	*Halocaridina rubra*	16,065	DQ917432
Caridea	Atyidae	*Halocaridinides fowleri*	15,997	NC035412
Caridea	Atyidae	*Neocaridina heteropoda*	15,558	MK907783
Caridea	Atyidae	*Neocaridina denticulata*	15,561	NC023823
Caridea	Atyidae	*Paratya australiensis*	15,990	NC027603
Caridea	Atyidae	*Stygiocaris lancifera*	15,787	NC035404
Caridea	Atyidae	*Stygiocaris stylifera*	15,812	KX844722
Caridea	Atyidae	*Typhlatya taina*	15,790	NC035399
Caridea	Atyidae	*Typhlatya pearsei*	15,798	NC035400
Caridea	Atyidae	*Typhlatya monae*	16,007	NC035405
Caridea	Atyidae	*Typhlatya mitchelli*	15,814	NC035403
Caridea	Atyidae	*Typhlatya miravetensis*	15,865	NC036335
Caridea	Atyidae	*Typhlatya iliffei*	15,926	NC035401
Caridea	Atyidae	*Typhlatya garciai*	15,318	NC035409
Caridea	Atyidae	*Typhlatya galapagensis*	16,430	NC035402
Caridea	Atyidae	*Typhlatya dzilamensis*	15,892	NC035408
Caridea	Atyidae	*Typhlatya consobrina*	15,758	NC035407
Caridea	Atyidae	*Typhlatya arfeae*	15,887	NC035410
Caridea	Atyidae	*Typhlopatsa pauliani*	15,824	NC035406
Caridea	Lysmatidae	*Exhippolysmata ensirostris*	16,350	MK681888
Caridea	Palaemonidae	*Exopalaemon carinicauda*	15,730	NC012566
Caridea	Palaemonidae	*Palaemon modestus*	15,736	MF687349
Caridea	Palaemonidae	*Palaemon gravieri*	15,735	KU899135
Caridea	Palaemonidae	*Palaemon capensis*	15,925	NC039373
Caridea	Palaemonidae	*Anchistus australis*	15,396	NC046034
Caridea	Palaemonidae	*Palaemon sinensis*	15,955	MH880828
Caridea	Palaemonidae	*Palaemon annandalei*	15,718	NC038117
Caridea	Palaemonidae	*Macrobrachium lanchesteri*	15,694	Nc012217
Caridea	Palaemonidae	*Macrobrachium rosenbergii*	15,964	NC012217
Caridea	Palaemonidae	*Macrobrachium nipponense*	15,806	HQ830201
Caridea	Palaemonidae	*Macrobrachium rosenbergii*	15,772	AY659990
Caridea	Pandalidae	*Chlorotocus crassicornis*	15,935	NC035828
Caridea	Pandalidae	*Pandalus borealis*	15,956	LC341266
Caridea	Pandalidae	*Heterocarpus ensifer*	15,939	MG674228
Caridea	Pandalidae	*Bitias brevis*	15,891	NC040856
Stenopodidea	Stenopodidae	*Stenopus hispidus*	15,528	NC018097
Stenopodidea	Spongicolidae	*Spongiocaris panglao*	15,909	NC038166

## Data Availability

The mitochondrial genome data has been submitted to NCBI GenBank under the following Accession Numbers MK681888.
